# *Listeria Monocytogenes*: A Model Pathogen Continues to Refine Our Knowledge of the CD8 T Cell Response

**DOI:** 10.3390/pathogens7020055

**Published:** 2018-06-16

**Authors:** Zhijuan Qiu, Camille Khairallah, Brian S. Sheridan

**Affiliations:** Department of Molecular Genetics & Microbiology, Center for Infectious Diseases, Stony Brook University, Stony Brook, NY 11790, USA; Zhijuan.Qiu@stonybrook.edu (Z.Q.); Camille.Khairallah@stonybrook.edu (C.K.)

**Keywords:** *Listeria monocytogenes*, CD8 T cells, dendritic cells, T cell activation, expansion, differentiation, contraction, and memory formation, resident memory T cells, CD8 T cell-mediated protective immunity, vaccine, cancer immunotherapy

## Abstract

*Listeria monocytogenes* (*Lm*) infection induces robust CD8 T cell responses, which play a critical role in resolving *Lm* during primary infection and provide protective immunity to re-infections. Comprehensive studies have been conducted to delineate the CD8 T cell response after *Lm* infection. In this review, the generation of the CD8 T cell response to *Lm* infection will be discussed. The role of dendritic cell subsets in acquiring and presenting *Lm* antigens to CD8 T cells and the events that occur during T cell priming and activation will be addressed. CD8 T cell expansion, differentiation and contraction as well as the signals that regulate these processes during *Lm* infection will be explored. Finally, the formation of memory CD8 T cell subsets in the circulation and in the intestine will be analyzed. Recently, the study of CD8 T cell responses to *Lm* infection has begun to shift focus from the intravenous infection model to a natural oral infection model as the humanized mouse and murinized *Lm* have become readily available. Recent findings in the generation of CD8 T cell responses to oral infection using murinized *Lm* will be explored throughout the review. Finally, CD8 T cell-mediated protective immunity against *Lm* infection and the use of *Lm* as a vaccine vector for cancer immunotherapy will be highlighted. Overall, this review will provide detailed knowledge on the biology of CD8 T cell responses after *Lm* infection that may shed light on improving rational vaccine design.

## 1. Introduction

*Listeria monocytogenes* (*Lm*) is a Gram-positive, facultatively anaerobic intracellular bacterium that can cause listeriosis. It is a foodborne pathogen and primarily affects pregnant women, immunocompromised individuals, the young, and the elderly but may also adversely affect otherwise healthy individuals during outbreaks. *Lm* infection of pregnant women can lead to infection of the fetus and result in fetal resorption, miscarriage or stillbirth, significantly contributing to the high mortality rate of *Lm* infections. Premature delivery and vertical transmission to the newborn are also serious complications associated with infection during pregnancy. Infections of susceptible populations may result in sepsis, meningitis, and encephalitis, which could be lethal. However, infections of otherwise healthy individuals typically lead to gastroenteritis. While rare, exposure to outbreak levels of *Lm* in healthy individuals could also be fatal. In the United States, according to the Centers for Disease Control and Prevention and a recent report conducted by United States Department of Agriculture, *Lm* is the third leading cause of deaths resulting from foodborne diseases and costs approximately 2.6 billion dollars annually, ranking it the third most among foodborne diseases in economic burden [1,2,3]. *Lm* infects humans by invading the intestinal epithelium after consumption of contaminated food. The bacterial surface protein internalin A (InlA) promotes the invasion of human intestinal epithelium by binding to E-cadherin (Ecad), an adhesion molecule expressed by intestinal epithelial cells [4]. However, InlA does not recognize murine Ecad, and *Lm* fails to invade mouse intestines efficiently [5], limiting the use of mice as a model for oral *Lm* infection of humans. Therefore, the understanding of *Lm* pathogenesis and the immune response to *Lm* infection has predominantly been obtained after intravenous (i.v.) infection of mice. As such, this review will primarily summarize the knowledge originating from studies performed in i.v. *Lm* infection models. The more recent generation of transgenic mice expressing a human Ecad or a humanized murine-Ecad and a murinized *Lm* strain containing mutations in the InlA protein that allow efficient invasion of murine intestines that may be coupled with a natural feeding infection provides more relevant mouse models for oral *Lm* infection or vaccination of humans [6,7,8,9,10]. Thus, this review will also discuss knowledge gained from oral *Lm* infection using these mouse models when available. 

**Figure 1:**
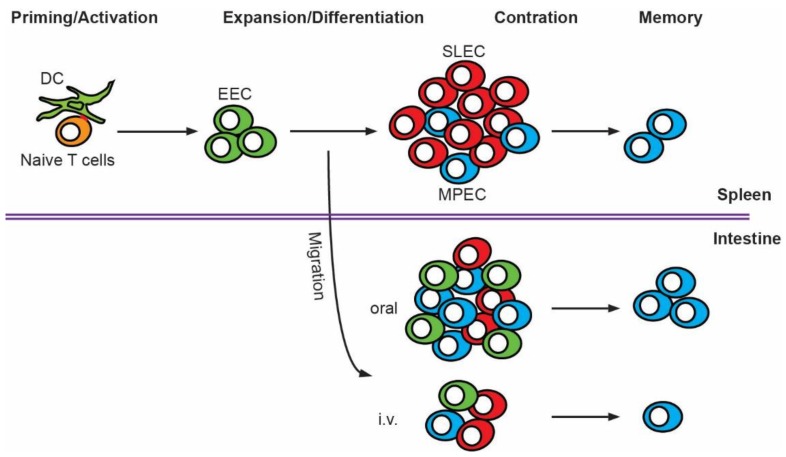
Schematic for the CD8 T cell response to *Lm* infection. The CD8 T cell response to *Listeria monocytogenes (Lm)* infection can be characterized by several major phases: (1) priming and activation; (2) clonal expansion and differentiation; (3) contraction; and (4) memory. Dendritic cells (DC) acquire *Lm* and present antigen to naïve CD8 T cells to activate them. Activated CD8 T cells subsequently undergo clonal expansion and differentiation. CD8 T cells first differentiate into early effector cells (EEC), which may become short-lived effector cells (SLEC) or memory precursor effector cells (MPEC). Following the peak of clonal expansion and pathogen clearance, the majority of effector CD8 T cells die during contraction. The remaining effector cells survive to form a long-lived memory population that can provide protection to subsequent challenges. During expansion and differentiation, effector CD8 T cells migrate to the intestine where they form resident memory CD8 T cells. Effector CD8 T cells differentiate mostly into SLEC in the spleen, while they are skewed towards EEC and MPEC in the intestine. The magnitude and differentiation pattern of effector CD8 T cells in the intestine differ after intravenous (i.v.) and oral *Lm* infection.

Innate inflammatory responses are critical for host defense against *Lm* infection. A hierarchical recruitment and activation of innate immune cells such as dendritic cells (DC) and inflammatory monocytes to the foci of infection coupled with interleukin (IL)-12, IL-18, interferon (IFN)-γ and tumor necrosis factor (TNF)-α production are essential for the early control of *Lm* infection [11]. However, sterilizing immunity to *Lm* infection requires T cells [12,13,14]. CD8 T cells, along with CD4 T cells and γδ T cells collaborate to provide optimal protection against *Lm* infection [9,13,14,15]. Extensive research has been carried out in the past three decades to broaden our understanding of T cell responses to *Lm* infection. *Lm* is also a model pathogen to study T cell biology in general because of its ability to induce robust T cell responses that are readily tractable during all phases of the adaptive response [16,17]. This review will focus on the CD8 T cell response to *Lm* infection, which can be characterized by four phases: (1) priming and activation; (2) clonal expansion and differentiation; (3) contraction; and (4) memory formation (Figure 1). Details of each phase of the CD8 T cell response to *Lm* infection will be discussed. Specifically, the role of dendritic cell subsets in acquiring and presenting *Lm* antigens to CD8 T cells and events that occur during CD8 T cell priming and activation will be addressed. Signals that regulate CD8 T cell expansion, differentiation and contraction during *Lm* infection will be explored. The formation of memory CD8 T cell subsets in the circulation and in the intestine will be analyzed. Additionally, the comparison of the CD8 T cell response after i.v. versus oral *Lm* infection will be included. Finally, CD8 T cell-mediated protective immunity against *Lm* infection and the use of *Lm* as a vaccine vector for cancer immunotherapy will be highlighted.

## 2. *Listeria Monocytogenes (Lm)* Acquisition and Presentation by Dendritic Cells (DC)

After i.v. infection, *Lm* directly enters the blood circulation and rapidly arrives in the marginal zone of the spleen, where it is taken up by macrophage receptor with collagenous structure (MARCO)^+^ marginal zone macrophages (MZM) and CD169^+^ marginal metallophilic macrophages (MMM) [18,19,20]. These macrophages are thought to be crucial for the early control of *Lm* infection as shown by studies using low dose clodronate liposomes to deplete both macrophage subsets [18]. A recent study using transgenic mice expressing human diphtheria toxin receptor under the control of the *Cd169* promoter to selectively deplete CD169^+^ MMM demonstrated that they are the primary line of defense against *Lm* infection [20]. In the absence of CD169^+^ MMM, *Lm* spreads to the red pulp of the spleen, where it rapidly replicates leading to systemic dissemination [20]. CD169^+^ MMM initially contain *Lm* in the marginal zone, and *Lm* is subsequently transported to the T cell zone of the white pulp [21,22]. Several sophisticated studies have shown that basic leucine zipper ATF-like transcription factor 3 (*Batf3)*-dependent CD8α^+^ DC are responsible for shuttling *Lm* to T cell zones of the white pulp [22,23,24]. The translocation of *Lm* to the T cell zone is a prerequisite for the establishment of a productive infection and the initiation of antigen presentation to CD8 T cells [22,23,24]. *Lm* appears to be targeted to *Batf3*-dependent CD8α^+^ DC by its association with platelets that is dependent on complement C3 and platelet receptor glycoprotein membrane complex Ib (GPIb) [25]. However, a recent study identified a new pathway in which *Lm* may be targeted to *Batf3*-dependent CD8α^+^ DC early after i.v. infection. CD169^+^ MMM were visualized acquiring *Lm* in the marginal zone and trans-infecting *Batf3*-dependent CD8α^+^ DC to initiate *Lm* transit to the T cell zone [20]. Thus, in the absence of CD169^+^ MMMs, *Batf3*-dependent CD8α^+^ DC failed to transport *Lm* to the T cell zone [20]. Whether platelet association directly targets *Lm* to *Batf3*-dependent CD8α^+^ DC or indirectly through CD169^+^ MMMs remains to be elucidated. In the absence of *Batf3*-dependent CD8α^+^ DC, *Lm* was unable to establish a productive infection in the T cell zone as they were confined to the marginal zone and rapidly cleared by macrophages [20,24]. As such, CD8 T cell responses were also significantly impaired [24]. This impairment could be rescued by increasing infectious dose or adoptive transfer of *Lm*-infected bone marrow-derived macrophages [23,24], suggesting that *Batf3*-independent DC are also capable of priming CD8 T cell responses to i.v. *Lm* infection. However, under normal physiological conditions, *Batf3*-dependent CD8α^+^ DC appear to play a central role in the activation of CD8 T cells, which has also been corroborated by in vitro studies showing that CD8α^+^ DC are more effective than CD11b^+^ DC at eliciting CD8 T cell responses to *Lm* [26]. In addition to their role in transporting *Lm* to the T cell zone and activating CD8 T cells, a new study demonstrated that *Batf3*-dependent CD8α^+^ DC are a vital source of IL-18, which subsequently licenses Natural Killer (NK) cells to produce IL-10 [27]. As NK cell-derived IL-10 promotes susceptibility to *Lm* infection [28], this new study provides an additional mechanism that contributes to the resistance of mice deficient in *Batf3*-dependent CD8α^+^ DC to *Lm* infection. 

After oral infection, *Lm* invades the gut epithelium and disseminates to the draining mesenteric lymph nodes (MLN), a primary site of T cell priming in response to intestinal pathogens. Whether *Lm* disseminates to the MLN extracellularly or intracellularly remains to be elucidated. While mechanistic in vivo insights of *Lm* dissemination is lacking, intracellular localization and replication appears essential for *Lm* dissemination to the MLN [29], suggesting that *Lm* may disseminate to the MLN intracellularly. Both intestinal CD103^+^ DC and C-X_3_-C motif chemokine receptor 1 (CX_3_CR1)^+^ mononuclear phagocytes (MP) can sample antigens from the lumen and migrate to the MLN in a C-C motif chemokine receptor (CCR)7-dependent manner [30,31,32,33,34]. CX_3_CR1^+^ MP are located close to the intestinal epithelium while CD103^+^ DC reside deeper in the lamina propria (LP) [33]. CX_3_CR1^+^ MP have been reported to capture luminal bacteria by extending transepithelial dendrites into the lumen [30]. CD103^+^ DC can be recruited to the intestinal epithelium in response to enteropathogen infection and can also capture luminal bacteria using transepithelial dendrites [32]. CD103^+^ DC may also acquire low molecular weight soluble luminal antigen from small intestine goblet cells through goblet cell-associated antigen passages [34]. A collaboration between CX_3_CR1^+^ MP and CD103^+^ DC has also been reported, where CX_3_CR1^+^ MP initially acquire luminal antigens for transfer to CD103^+^ DC [35]. *Lm* appears to preferentially target luminally accessible Ecad on goblet cells and utilizes the transcytosis pathway to gain access to the lamina propria [36], implying that CD103^+^ DC may play a direct role in the acquisition of *Lm* and the transportation of *Lm* to the MLN. CD103^+^ DC can efficiently generate CCR9^+^ α_4_β_7_^+^ gut-tropic effector CD8 T cells after oral administration of antigen [37]. However, CD103^+^ DC consist of two distinct subsets, interferon regulatory factor (IRF)8-dependent CD11b^−^ CD103^+^ DC and IRF4-dependent CD11b^+^ CD103^+^ DC [38,39,40]. Whether CD11b^−^ CD103^+^ DC, CD11b^+^ CD103^+^ DC, or both are important for carriage of *Lm* to MLN and subsequent T cell priming is unresolved. IRF4-dependent CD11b^+^ CD103^+^ DC play a critical role in driving mucosal T helper (Th)17 responses [40], while IRF8-dependent CD11b^−^ CD103^+^ DC induce a Th1 response [41]. *Lm* can induce either Th1 or Th17 responses dependent on the route of infection. While i.v. *Lm* infection induces Th1 cells, intranasal *Lm* infection induces Th17 cells [42]. Our recent study demonstrated that a Th1 response is primarily induced after oral *Lm* infection [15], suggesting the involvement of CD11b^−^ CD103^+^ DC but not CD11b^+^ CD103^+^ DC in the induction of T cell responses after oral *Lm* infection. However, further work needs to determine whether the acquisition and transit of *Lm* is uncoupled from T cell priming, in which case one DC subset may acquire and transport *Lm* to the MLN and another DC subset may prime T cells and generate gut-tropic effector CD8 T cells after oral *Lm* infection.

## 3. T Cell Priming and Activation

Circulating naïve CD8 T cells enter secondary lymphoid organs where they quickly survey DC before forming prolonged stable conjugates with DC presenting their cognate antigens [43]. During i.v. *Lm* infection, antigen-specific CD8 T cells form clusters with DC at the borders of the T and B cell zones in the spleen [44]. Immunological synapses are organized at the interfaces of T cells and DC with apparent polarization of T cell receptor (TCR) and CD8 co-receptor, indicating the initiation of T cell activation. Antigen-specific CD8 T cells increase size, downregulate CD62L, and upregulate CD11a, programmed cell death protein 1 (PD-1) and CD69 [44]. Following priming and activation by DC, antigen-specific CD8 T cells migrate to the T cell zones, where they undergo extensive proliferation before exiting the white pulp via bridging channels for entry into the red pulp and exit from the spleen. For i.v. *Lm* infection, antigen-specific CD8 T cell responses peak around 7–8 days post infection (dpi) [45,46]. Presumably, during oral infection with mouse-adapted *Lm*, CD8 T cells in the MLN undergo a similar process including initial priming and activation by DC followed by vigorous proliferation in the T cell zones and rapid egress from the MLN. While *Lm* enter the spleen within minutes after i.v. infection, *Lm* access to the MLN from the gut is delayed. Accordingly, antigen-specific CD8 T cell responses peak at around 8–9 dpi after oral *Lm* infection [47,48].

CD8 T cell priming and activation by DC is a crucial step that ensures the generation of functional effector T cells critical for pathogen clearance by eliminating intracellular reservoirs of infected cells. During i.v. infection, efficient CD8 T cell priming and activation occur after infection with live *Lm* but not administration of heat-killed *Lm* (HK*Lm*) [49,50]. Following HK*Lm* administration, CD8 T cells undergo poor proliferation and fail to upregulate activation markers such as CD69 and PD-1 [49,50]. These CD8 T cells also exhibit limited cytolytic activity and impaired cytokine production [49,50]. As a result, immunization with HK*Lm* does not induce protective immunity [49,51,52,53]. Multiple mechanisms may account for inefficient CD8 T cell induction after HK*Lm* administration. CD169^+^ MMM in the marginal zone of the spleen appear to be the primary cellular niche for *Lm* early after i.v. infection [20]. DC may directly phagocytose *Lm* in the marginal zone or indirectly acquire *Lm* from CD169^+^ MMM [19,20]. The latter requires recruitment of DC to infected CD169^+^ MMM, which is dependent on *Lm* invasion of the cytosol [20]. HK*Lm* fails to escape the phagolysosome and is unable to access the cytosol [54]. Therefore, DC may not acquire sufficient antigen after HK*Lm* administration, impairing their ability to induce a robust CD8 T cell response. In addition, while live *Lm* is rapidly transported to the T cell zone by DC [22,23,24], HK*Lm* remains in the marginal zone [55], suggesting that DC are unable to carry HK*Lm* to the T cell zone to activate T cells after HK*Lm* administration. Indeed, CD8 T cell-DC cluster formation was not observed after HK*Lm* administration [50]. Finally, HK*Lm* infection induced low levels of the costimulatory molecules CD80 and CD86 on DC [55], and this was independent of the amount of *Lm* uptake by DC suggesting an intrinsic defect associated with HK*Lm* [54]. CD28-mediated signals delivered by DC expressed CD80 and CD86 are important for CD8 T cell activation and expansion after *Lm* infection [56]. Furthermore, HK*Lm* fails to induce IFN-α/β production from DC [54]. IFN-β production from live *Lm*-infected DC induces CD69 expression on CD8 T cells and promotes CD8 T cell proliferation after antigen stimulation [54]. These studies suggest that HK*Lm* is unable to induce fully activated DC that can efficiently prime CD8 T cells. Collectively, these studies indicate that CD8 T cell priming and activation by DC after i.v. *Lm* infection is a multifaceted process involving DC acquisition of *Lm* that is capable of phagolysosomal escape followed by adequate DC maturation and efficient migration to the T cell zone.

The acquisition of *Lm* by DC is distinct after i.v. and oral infection. Compared to splenic DC, LP DC in steady state express higher levels of CD86, suggesting that they are more mature during homeostasis [57] and may have a lower activation threshold. Moreover, LP DC constitutively express CCR7 and readily migrate to the MLN upon antigen uptake [57]. Compared to splenic DC, LP DC selectively induce gut-homing receptor α_4_β_7_ and CCR9 expression on CD8 T cells [37], which has a profound impact on the tropism of CD8 T cells. However, whether CD8 T cells are primed and activated differently by DC after oral *Lm* infection and how that will impact their expansion, contraction, differentiation and memory formation are not well understood. 

## 4. T Cell Expansion, Differentiation and Contraction

Naïve antigen-specific CD8 T cells, present at very low frequencies (~80–1200 cells per mouse), undergo rapid and massive clonal expansion and development of effector functions after priming and activation by DC. A large population of effector cells are mobilized into the blood and migrate to sites of infection to eliminate intracellular pathogens by inducing cytolysis of infected cells. Effector CD8 T cells also produce potent anti-pathogen cytokines to aid in the resolution of infection [58,59]. Following the peak of clonal expansion and pathogen clearance, antigen-specific effector CD8 T cells undergo extensive contraction, during which most effector cells (90–95%) rapidly die through apoptosis restoring homeostasis of the immune system. The remaining effector cells survive to form a long-lived self-renewing memory population that can provide life-long protection against reinfection [60]. Effector CD8 T cells that are fated to die during contraction and those that possess memory potential can be identified based on the dichotomous expression of killer cell lectin-like receptor G1 (KLRG-1) and IL-7Rα (CD127) [61,62,63,64,65]. Naïve CD8 T cells express CD127 but not KLRG-1 [61,62,64,65,66]. Within the first few days after antigen encounter, CD8 T cells downregulate CD127 and form a plastic population of CD127^−^ KLRG-1^−^ early effector cells (EEC) [46,67]. EEC can upregulate KLRG1 to differentiate into CD127^−^ KLRG-1^+^ short-lived effector cells (SLEC) or reexpress CD127 to differentiate into CD127^+^ KLRG-1^−^ memory precursor effector cells (MPEC). SLEC are terminally differentiated and undergo apoptosis during immune contraction, while MPEC have long-lived potential and survive into self-maintaining memory cells. In some circumstances, a subset of cells expressing both KLRG-1 and CD127 develop, but their developmental pathway and immunological role are less clear [46,68].

Antigen-specific CD8 T cell expansion and contraction after i.v. *Lm* infection is instructed during priming [69,70]. However, manipulation of the infection to influence the amount and duration of antigen and inflammation by using antibiotic treatment, attenuated strains or different doses of *Lm* can greatly impact these processes. Increasing the infectious dose can increase antigen-specific CD8 T cell expansion and the magnitude of the peak response, but it does not appear to affect the onset or early kinetics of contraction [70]. Shortening the length of infection by antibiotic treatment early after infection decreases the magnitude of antigen-specific CD8 T cell expansion [69,70,71,72]. However, the onset of T cell contraction seems to be predominately influenced by the peak of bacterial burden or antigenic load but not the length of infection [72]. Infection with a highly attenuated actin assembly-inducing protein (ActA)-deficient *Lm* that is not able to spread from cell to cell intracellularly leads to a quicker peak of bacterial load and an accelerated antigen-specific CD8 T cell response with earlier onset of contraction [72]. Continuous treatment of animals with antibiotics before and throughout the infection also significantly impairs the expansion of antigen-specific CD8 T cells [71,73]. Intriguingly, antigen-specific CD8 T cells generated in these antibiotic treated animals do not undergo contraction, leading to a normal and functional memory population despite a substantially reduced effector response. The lack of contraction is thought to be associated with decreased inflammation caused by continuous antibiotic treatment. In such environments, antigen-specific CD8 T cells do not upregulate KLRG-1 to differentiate into SLEC. Instead, they upregulate CD127 and become MPEC that survive and form memory. These studies demonstrate that the inflammatory environment regulates T cell memory differentiation.

CD8 T cell memory differentiation is a continuous process; however, fate decisions occur early during the effector phase at the EEC stage and are largely dictated by the nature of the pathogen and environmental conditions they induce [46,74,75]. After i.v. *Lm* infection, EEC predominately give rise to SLEC in the spleen, leading to a dominant SLEC population (~75%) with few EEC (~10%) and MPEC (~5%) [46,74,75]. In comparison, after i.v. vesicular stomatitis virus (VSV) infection, some EEC stay undifferentiated and those that differentiate form both SLEC and MPEC in the spleen, resulting in roughly comparable populations of EEC (~35%), SLEC (~35%) and MPEC (~25%) [46,74,75]. The differentiation pattern seen in i.v. VSV infection has also been observed in intranasal influenza A virus infection and vaccinia virus infection via skin scarification [74]. This distinct pathogen-induced differentiation pattern was observed at both the population and single-cell levels [46,74,75]. Moreover, both i.v. and oral *Lm* infection induced a similar pattern in the spleen with a heavily skewed SLEC population, suggesting that the differentiation pattern of EEC appears pathogen driven [75]. Interestingly, while EEC appear committed to either a SLEC or MPEC fate during priming, they retain plasticity to respond to changing environmental cues [74]. For example, EEC from *Lm*-infected mice mainly differentiated into SLEC when transferred into naïve mice. However, transfer of *Lm*-elicited EEC into a mouse infected with VSV expressing the same cognate antigen resulted in a differentiation pattern resembling that observed after VSV infection. Thus, EEC display some level of superficial commitment to a specific lineage based on early signals while maintaining a degree of plasticity to respond appropriately to changing inflammatory cues. This can be further observed in vivo at the single cell level [75]. Unique clones of naïve CD8 T cells that differentiate into effector CD8 T cells with bias to a single developmental pathway can be heavily skewed towards a different development pathway by tissue-specific environments. At the peak of the CD8 T cell response after oral *Lm* infection, effector CD8 T cells that arose from a single naïve T cell comprised mostly SLEC in the spleen but were heavily skewed towards MPEC and EEC once they migrated into the intestinal epithelium despite being progeny of an identical parent [48,75]. Thus, differentiation patterns can be heavily influenced by the distinct local environments of nonlymphoid tissues.

Pathogen-induced inflammation, when coupled with antigen, critically regulates SLEC and MPEC differentiation [74,76]. Reduced inflammation favors MPEC differentiation, whereas increased inflammation promotes SLEC differentiation [64,71]. I.v. *Lm* or VSV infection induce distinct inflammatory environments leading to unique differentiation patterns of their effector populations [46]. I.v. *Lm* infection elicits IL-12, IFN-β and IFN-γ, while VSV infection fails to induce these cytokines. IL-12 signaling promotes antigen-specific CD8 T cell expansion and SLEC differentiation in i.v. *Lm* infection and CD8 T cells lacking IL-12 receptor have impaired expansion and fail to differentiate into SLEC [46,77]. Mechanistically, IL-12 induces the transcription factor T-bet, which is necessary and sufficient to drive SLEC differentiation [64,78]. IFN-γ signaling can also promote SLEC differentiation following i.v. *Lm* infection. Antigen-specific CD8 T cells in IFN-γ deficient mice have increased CD127 expression [71]. However, IFN-γ does not induce SLEC differentiation directly [76]. Instead, it influences SLEC differentiation indirectly by promoting IL-12 production [76]. Type I interferon signaling has also been shown to promote antigen-specific CD8 T cell expansion and SLEC differentiation after i.v. *Lm* infection. CD8 T cells lacking type I interferon receptor fail to undergo robust expansion and cannot efficiently generate SLEC [46]. CD8 T cells lacking both IL-12 receptor and type I interferon receptor have a more profound defect in expansion and SLEC differentiation [46], suggesting that IL-12 and type I interferon play non-redundant roles in driving effector T cell expansion and SLEC differentiation. Overall, i.v. *Lm* infection favors SLEC differentiation by inducing an environment that promotes SLEC formation.

During i.v. *Lm* infection, both SLEC and MPEC undergo contraction; however, SLEC contract approximately 10 times more than MPEC [79]. The survival of MPEC is primarily dependent on IL-7, but IL-15 may also contribute to MPEC survival in some contexts [61,62,79]. Both IL-7 and IL-15 promotes cell survival in part by upregulating the expression of the anti-apoptotic molecule Bcl-2 [61,62,80,81], although these cytokines are not interchangeable. While administration of exogenous IL-7 or IL-15 during the contraction phase promotes the survival of MPEC [79], the presence of IL-7 but not IL-15 appears necessary, as MPEC fail to survive in the absence of IL-7 but they survive similarly in the presence or absence of IL-15 [61,62,64]. Thus, while IL-15 may promote MPEC survival, IL-7 is necessary for MPEC survival. The expression of CD127 allows MPEC to survive and form long-lived memory cells in the presence of IL-7; however, it is not sufficient to instruct memory formation as forced CD127 expression on SLEC does not save them from death [82,83]. As SLEC do not express CD127, their survival during contraction is predominantly dependent on IL-15 [64,79,80,81]. In the absence of IL-15, SLEC contract more rapidly, indicating IL-15 promotes some level of SLEC survival during contraction [64,80,81]. However, the ability to sense IL-15 is not sufficient for their long-term survival as SLEC still contract ~20-fold after i.v. *Lm* infection [79]. The massive contraction of SLEC is induced by transforming growth factor-β (TGF-β), which is upregulated after i.v. *Lm* infection and selectively promotes the apoptosis of SLEC during clonal expansion and contraction by dampening B-cell lymphoma (Bcl)-2 expression [81]. While both SLEC and MPEC express TGF-β receptor, IL-7 but not IL-15 seems to be able to overcome the apoptotic effect induced by TGF-β. Thus, TGF-β and IL-15 exert opposite roles in controlling the fate of SLEC after i.v. *Lm* infection.

Oral *Lm* infection induces similar kinetics of T cell expansion and contraction and a similar differentiation pattern in the spleen as i.v. infection, with the exception that antigen-specific CD8 T cells peak one day later after oral infection [45,46,47,48,75]. However, as discussed above the differentiation pattern can be profoundly impacted by the tissue-specific environment [75]. While antigen-specific CD8 T cells are largely SLEC in the spleen after oral *Lm* infection, the population rapidly shifts to MPEC in the intestine [48]. It appears SLEC undergo accelerated apoptosis in response to TGF-β signaling in the intestine, leading to the rapid accumulation of MPEC. This suggests that antigen-specific CD8 T cells in the intestine are more susceptible to TGF-β-induced apoptosis or that TGF-β signaling is more abundant in the intestine. Future studies are required to elucidate the detailed mechanisms involved in intestinal CD8 T cell differentiation. 

## 5. T Cell Memory Formation

After pathogen clearance, MPEC that survive the contraction phase give rise to long-lived memory cells. Memory CD8 T cells are heterogenous and have been traditionally divided into central memory T (T_CM_) cells and effector memory T (T_EM_) cells based on their migratory patterns [84]. T_CM_ cells express lymph node homing receptors CD62L and CCR7 and circulate between the bloodstream and secondary lymphoid organs. T_EM_ cells lack these receptors and circulate through the bloodstream, permissive non-lymphoid tissues and secondary lymphoid organs. I.v. *Lm* infection induces rapid generation of CD62L^+^ T_CM_ cells [85]. CD62L^+^ cells emerge in a subset of MPEC at the peak of the T cell response and gradually increase over time [85]. The entire antigen-specific CD8 T cell population gradually shifts from CD62L^−^ T_EM_ cells to CD62L^+^ T_CM_ cells. A linear T_EM_ → T_CM_ differentiation pathway had been proposed, in which T_EM_ cells are transitional and give rise to T_CM_ cells [86]. However, this does not appear to be the dominant pathway under normal physiological conditions [87]. CD62L^−^ T_EM_ cells generated under abnormally elevated precursor frequencies are not fully committed and capable of re-expressing CD62L and converting to CD62L^+^ T_CM_ cells. However, under physiological conditions with low precursor frequencies of naïve antigen-specific CD8 T cells, or in adoptive transfer systems where small numbers of naïve TCR transgenic CD8 T cells are used, CD62L^−^ T_EM_ and CD62L^+^ T_CM_ cells appear as distinct and stable lineages that develop independently without interconversion [87]. The gradual shift of the antigen-specific memory CD8 T cell population from CD62L^−^ T_EM_ cells to CD62L^+^ T_CM_ cells occurs due to a higher proliferative capacity of CD62L^+^ T_CM_ cells leading to their preferential accumulation over time [59,85,87]. Overall, CD62L expression and T_EM_/T_CM_ lineage commitment is largely influenced by the initial frequency of naïve antigen-specific CD8 T cells [59,87,88]. T_EM_/T_CM_ lineage decision occurs during the primary immune response [87]. It is generally believed that weak stimulation favors the generation of CD62L^+^ T_CM_ cells, while strong stimulation is required for CD62L^−^ T_EM_ cell generation. Indeed, limiting antigen availability and/or inflammation during i.v. *Lm* infection by blocking antigen presentation or shortening the infection promotes CD62L^+^ T_CM_ cell development [85,89]. Both T_EM_ and T_CM_ cells are capable of proliferating, producing cytokines such as IFN-γ and TNF-α and acquiring cytotoxicity upon antigenic stimulation, although T_CM_ cells have greater proliferative capacity and can produce IL-2 [86]. However, their protective capacity for challenge infections is greatly dependent on the characteristics of both the pathogen (i.e., site where the pathogen replicates and activation of T cells occurs) and memory subset (i.e., proliferative capacity and migratory preference) [90,91]. In i.v. *Lm* challenge infection, both T_EM_ and T_CM_ cells mount recall responses and contribute to protective immunity, with T_EM_ cells providing superior protection [90,92,93]. 

The identification of tissue-resident memory T (T_RM_) cells was a breakthrough in the field of memory CD8 T cells [48,94,95,96,97] that substantially improved our understanding of memory CD8 T cell subsets and their protective functions in tissues. Contrary to circulating T_EM_ and T_CM_ cells, T_RM_ cells represent a subset of memory T cells that are self-maintained in tissues without the need for replenishment from the circulation. They are phenotypically, functionally, transcriptionally, and metabolically distinct from T_EM_ and T_CM_ cells [98,99,100,101]. T_RM_ cells do not express CD62L and CCR7; instead, they express CD69, which provides a mechanism promoting their retention in tissues [102,103]. CD69 physically interacts with sphingosine-1-phosphate receptor 1 (S1PR1) and inhibits the S1PR1-mediated egress of CD8 T_RM_ cells from tissues [104,105]. Additionally, some T_RM_ cells also express CD103, which binds Ecad expressed by epithelial cells in barrier tissues and plays an important role in the retention of CD8 T_RM_ cells in barrier tissues [98,106,107]. CD8 T_RM_ cells have been shown to play a critical role in protective immunity against infections and cancers [48,94,95,97,108,109,110]. They are pre-positioned in the tissue to respond immediately to pathogen re-encounter and mediate protective immunity by direct lysis of infected cells or by activating innate immune cells and recruiting circulating memory T cells through the release of cytokines IFN-γ, IL-2 and TNFα [111,112,113]. Recent studies using an oral infection model of *Lm* demonstrated the robust induction of antigen-specific CD8 T cell responses in the intestine [47,48,114]. These intestinal antigen-specific CD8 T cells quickly adopted an MPEC phenotype and upregulated CD69 and CD103 expression, indicating rapid generation of CD8 T_RM_ cells in the intestine 9 days after oral *Lm* infection [48]. The expression of CD69 and CD103 was exclusively confined to MPEC, supporting the notion that CD8 T_RM_ cells are derived from MPEC. In this model, CD103 expression promoted the accumulation but not retention of antigen-specific CD8 T cells in the intestinal epithelium [48]. As CD103 also binds Ecad expressed by intestinal epithelial cells, it is possible that CD103 promotion of intestinal accumulation early after infection is due to the nature of Ecad-mediated *Lm* entry into the intestinal epithelium, and this topic needs further exploration. The rapid generation and maintenance of CD69^+^ CD103^+^ CD8 T_RM_ cells in the intestine after oral *Lm* infection is critically regulated by TGF-β signaling. In the absence of TGF-β signaling, antigen-specific CD8 T cells migrated to the intestine efficiently but failed to become CD69^+^ CD103^+^ CD8 T_RM_ cells and were not maintained in the intestine [48]. These intestinal CD8 T_RM_ cells established early after primary oral *Lm* infection provided optimal protection against secondary oral *Lm* infection [48]. Compared to oral *Lm* infection, i.v. *Lm* infection induced a significantly smaller population of antigen-specific CD8 T cells in the intestine and these CD8 T cells were inefficient at rapidly differentiating into CD69^+^ CD103^+^ CD8 T_RM_ cells, suggesting that the route of infection greatly impacts memory CD8 T cell responses in the intestine (our unpublished data). The migration of antigen-specific CD8 T cells to the intestine is controlled by gut-homing receptors α_4_β_7_ and CCR9 [115,116]. CD8 T cells induced after oral *Lm* infection likely express higher levels of α_4_β_7_ and CCR9 and migrate more efficiently to the intestine than those induced after i.v. infection as LP DC but not splenic DC selectively instruct CD8 T cells to upregulate α_4_β_7_ and CCR9 expression [37], which could contribute to the difference in the magnitude of antigen-specific CD8 T cell responses in the intestine after i.v. and oral infection. However, how infection route regulates the differentiation of CD8 T_RM_ cells in the intestine is unclear. Oral infection likely induces a distinct intestinal environment that may impact in situ differentiation of T_RM_ cells. Overall, i.v. and oral *Lm* infection appears to induce distinct CD8 cell responses in the intestine, which may greatly impact CD8 T_RM_ cell-mediated immunity. Future studies are required to evaluate the mechanisms governing the induction of superior gastrointestinal CD8 T cell responses after oral infection, which will improve our knowledge of mucosal T cell immunity and provide valuable insights into vaccine design. 

## 6. CD4 T Cell Help

The role of CD4 T cell help in regulating CD8 T cell responses has a long and often contradictory history [117], which is well documented after i.v. *Lm* infection. Lack of CD4 T cell help has been reported to impair the primary CD8 T cell response [118], the maintenance of memory CD8 T cells [119,120], or the recall CD8 T cell response [118,121]. Alternatively, CD4 T cell help has also been reported to be not critical for the primary CD8 T cell response [49,122], the maintenance of memory CD8 T cells [122], or the recall CD8 T cell response [49,122]. Traditionally, CD4 T cells were envisioned to provide help to CD8 T cells through multiple mechanisms such as activation of antigen presenting cells through CD40L/CD40 interaction (indirect help) or the secretion of IL-2 (direct help). Recently, CD4 T cell help has also been shown to facilitate migration of CD8 T cells into non-lymphoid tissues [123,124]. Whether CD4 T cell help to CD8 T cells during i.v. *Lm* infection is through CD40L/CD40 interaction is also controversial. While some studies showed that the CD40L–CD40 pathway was not required during the primary or recall CD8 T cell response [122,125,126], others showed that CD40L/CD40 interaction was required for the recall CD8 T cell response [63,118]. However, CD40L/CD40 interaction may provide help to CD8 T cells independently of CD4 T cells [118]. More recently, studies showed that CD4 T cell help induced the expression of CD25 by antigen-specific CD8 T cells, which was required for the optimal SLEC development and effector CD8 T cell expansion in response to IL-2 [127]. Studies further showed that memory CD8 T cells generated in the absence of CD25-mediated signals were able to mount a robust recall response [127], suggesting that CD4 T cell help and IL-2 signaling through CD25 controls the expansion and differentiation of effector CD8 T cells during the primary response but not the recall response.

During oral *Lm* infection, CD4 T cell help appears to be more important for CD8 T cell response in the intestinal tissues than the spleen and liver during primary response [47], suggesting that CD4 T cells may regulate CD8 T cell responses in a tissue-specific manner. Furthermore, CD4 T cells likely provide help to CD8 T cells through CD40L/CD40 interaction [47]. However, whether CD4 T cell help also regulates the maintenance of memory CD8 T cells and the recall CD8 T cell response after oral *Lm* infection is unclear.

## 7. CD8 T Cell-Mediated Protective Immunity against *Lm* Infection

Once *Lm* enters the host cell, it is able to use its surface protein ActA to induce actin polymerization and propel itself within the cell and spread to neighboring cells without exposure to the extracellular environment [4]. By remaining intracellular through its lifecycle, *Lm* can avoid humoral immunity. Thus, sterilizing immunity relies on inducing a robust cellular response [128]. CD8 T cells collaborate with CD4 T cells and γδ T cells to provide optimal protection against *Lm* infection [9,13,14,15]. The identification of CD8 epitopes from *Lm*-secreted proteins listeriolysin O (LLO) and invasion-associated protein p60 and the finding that CD8 T cells specific to either of these epitopes can provide protection against *Lm* infection led to the hypothesis that *Lm*-secreted proteins may be the most relevant antigens to prime CD8 T cells and to induce protective immunity against *Lm* infection [17,129,130,131,132,133]. Subsequent studies using recombinant *Lm* to express a secreted or non-secreted form of epitope derived from lymphocytic choriomeningitis or recombinant *Salmonella typhimurium* to express secreted or non-secreted forms of LLO and p60 suggested that both secreted and non-secreted epitope or protein can induce primary and secondary antigen-specific CD8 T cell responses [134,135,136]. However, these antigen-specific CD8 T cells provide protection against *Lm* expressing the secreted antigen but not against *Lm* expressing a non-secreted form of the same antigen [134,135,136]. Through ActA-mediated cell-to-cell spread, *Lm* can infect a variety of cells including phagocytic and non-phagocytic cells. In infected phagocytic cells, both secreted and non-secreted bacterial antigens can be presented on the cell surface, while in infected non-phagocytic cells, only secreted bacterial antigens can be displayed on the cell surface for immune surveillance [135]. Therefore, although phagocytic cells can present non-secreted antigens to CD8 T cells to prime them, CD8 T cells specific for non-secreted antigens do not recognize infected non-phagocytic cells and are unable to control listeriosis [135]. As maternal *Lm* infection can cause serious fetal or neonatal complications, developing prophylactic and therapeutic vaccines against listeriosis is an ongoing interest [137,138,139,140,141,142,143,144]. When designing CD8 T cell-based vaccines against listeriosis, it is important to keep it in mind that non-secreted antigens may not be relevant targets. 

Effective control of *Lm* infection by memory CD8 T cells in the organ where *Lm* invades may prevent further disseminating infection and limit more serious disease. *Lm* first invades the spleen or liver after i.v. infection and the intestine after oral infection. These organs contain distinct memory CD8 T cells with unique phenotypes, migratory properties, maintenance requirements, and functions [107,114,145]. Generally, memory CD8 T cells in the intestine express CD69 and some of them also express CD103, both of which are important mediators of tissue residency, while memory CD8 T cells in the spleen lack these markers [107,146]. Memory CD8 T cells in the spleen can circulate through lymphoid tissues or permissive non-lymphoid tissues dependent on their expression of the lymphoid homing receptor CD62L [107,145]. Memory CD8 T cells in the spleen but not intestine express CD122, IL-15 receptor beta, indicating distinct requirements of IL-15 for their maintenance [107,145,147]. Moreover, memory CD8 T cells in the intestine express higher granzyme B but lower IFN-γ, TNF-α and IL-2 compared to memory CD8 T cells in the spleen [107,145], suggesting functional tailoring to the unique tissue environment that may influence their contribution to protective immunity. These phenotypic and functional characteristics seem to be intrinsic to organ-specific environments, as CD8 T cells derived from a single naïve cell acquire different phenotypes when they enter the spleen or intestine [75]. However, the route of infection greatly impacts organ-specific memory CD8 T cell responses. Memory CD8 T cells are enriched in the spleen after i.v. *Lm* infection while they are enriched in the intestine after oral infection (our unpublished data). CD8 T_RM_ cells provide superior protection against pathogens invading the barrier tissues such as skin, female reproductive tract and lung [94,95,97,148]. Based on this evidence, it is plausible that memory CD8 T cells generated by oral *Lm* infection provides superior protection against *Lm* invading the intestine through contaminated food as more CD8 T_RM_ cells would be prepositioned at the location of invasion. Vice versa, it is likely that memory CD8 T cells generated by i.v. *Lm* infection can protect better against *Lm* invading the spleen as more memory CD8 T cells would be positioned in the spleen. Whether this same strategy would protect a fetus or neonate is unclear as the route of exposure and even the mediator of fetal resorption is less defined. For example, fetal exposure may occur through direct invasion of extracellular *Lm* via interaction with placental accessible Ecad [149] or via a trojan horse model where intracellular carriage by circulating immune cells mediates fetal exposure. Intriguingly, a recent study of pregnant mice indicated that CD8 T cells are required for *Lm*-induced fetal resorption [150]. Depletion of CD8 T cells, neutralization of T cell-derived IFN-γ, or blockade of decidual CD8 T cell accumulation protected against fetal wastage [150]. Thus, strategies aimed at preventing *Lm* invasion in the intestines may be the best approach to limit fetal and neonatal complications associated with *Lm* infection during pregnancy. Nevertheless, organ-specific CD8 T cell responses likely shape organ-specific protective immunity. When designing vaccines against listeriosis or other infections and malignancies, it is important to consider the potential benefits of organ-specific immunity.

## 8. Non-Classical H2-M3-Restricited CD8 T Cell Response

Although most studies focus on major histocompatibility complex (MHC) class Ia (H2-K)-restricted CD8^+^ T cells, another population of CD8^+^ T cells that recognizes secreted bacterial-derived N-formylated peptides presented by the nonclassical MHC class Ib molecule H2-M3 responds to *Lm* infection and distinctly contributes to anti-*Lm* immunity [151,152,153,154,155]. Despite the limited polymorphism of H2-M3 molecules, several distinct *Lm*-derived peptides containing N-formyl-methionine have been shown to induce CD8^+^ T cell responses [153,154,155], with fMIGWII being the major immunodominant epitope during *Lm* infection [156]. H2-M3-restricted CD8^+^ T cells express promiscuous antigen receptors which enable them to broadly recognize N-formylated peptides produced by *Lm* [157,158,159].

I.v. *Lm* infection results in the generation of both H2-K- and H2-M3-restricted CD8^+^ T cells; however, these populations differ in their expansion kinetics and memory potential. H2-M3-restricted T cells rapidly and robustly expand in the spleen of infected animals, peaking 2 to 3 days before and outnumbering *Lm*-specific H2-K-restricted CD8^+^ T cells during primary infection [156,160,161]. H2-M3-restricted CD8 T cells were functional, displayed high cytotoxic activity and secreted high levels of IFN-γ [161]. Correspondingly, H2-M3-restricted CD8 T cells contribute to protection early during primary *Lm* infection, at a time when *Lm*-specific H2-K-restricted CD8^+^ T cells have not substantially expanded [162]. Although both CD8 T cell populations establish phenotypically similar memory populations and express activation markers upon secondary exposure to *Lm* [163], only H2-K-restricted memory CD8 T cells dramatically expanded after reinfection [156,160,161]. However, the impaired recall of H2-M3-resticted CD8 T cells appears to be an indirect consequence of the presence of H2-K-restricted memory CD8 T cells. Indeed, an *Lm* challenge of mice previously immunized with DCs coated with fMIGWII peptide triggered a vigorous expansion of H2-M3-restricted CD8 T cells [164]. However, in this context, H2-M3-restricted memory CD8 T cells were incapable of providing protective immunity to *Lm* challenge infection [164]. Thus, H2-M3-restricted CD8 T cells form a distinct non-classical CD8 T cell population, whose primarily role is to provide protection early during primary infection enabling sufficient time for the induction of long-term protective H2-K-restricted CD8 T cells. Whether H2-M3-restricted CD8 T cells are induced after oral *Lm* infection has not been studied.

## 9. *Lm* as a Vaccine Vector for Cancer Immunotherapy

*Lm* has gained prominence as a potential vaccine vector for cancer immunotherapy for several reasons [165,166]. First, *Lm* displays tumor-homing properties and specifically establishes tropism in primary and metastatic tumors that may result in direct killing of tumor cells [166,167,168,169]. Second, *Lm* induces a strong innate inflammatory immune response that is key to the induction of potent adaptive immunity and the efficacy of *Lm* as a cancer vaccine vector [11,165,166]. Third, *Lm* elicits robust CD8 T cell responses. *Lm* is able to escape the phagolysosome to gain access to the cytosol of professional antigen-presenting cells where it secretes antigens into the cytosol that are rapidly degraded and efficiently delivered to the MHC class I pathway to activate CD8 T cells [170]. Moreover, recent studies suggested that *Lm*-derived antigens are processed and presented with greater efficiency compared to endogenously synthesized viral antigens [171], further supporting the use of *Lm* as a vaccine vector to induce potent CD8 T cell responses. Fourth, *Lm*-elicited CD8 T cells can overcome tolerance to tumor-associated antigens [172,173], providing the rationale for using *Lm* as a vaccine vector for cancer immunotherapy. Fifth, *Lm*-based cancer vaccines have been shown to reduce the number and the suppressive activity of regulatory T cells and myeloid-derived suppressor cells in the tumor microenvironment [174,175,176,177], adding another layer of efficacy for *Lm*-based cancer vaccines. Sixth, *Lm* vaccines may be repeatedly administered to increase efficacy as antibodies do not appear sufficient to prevent boosting [9]. Finally, *Lm* is relatively easy to manipulate and a variety of attenuated strains have been created, lessening safety concerns of *Lm*-based therapeutics [165,166]. Overall, the above features make *Lm* one of the most promising vaccine vectors for cancer immunotherapy and may also engender *Lm*-based vaccines to pathogens that have proven difficult to immunize against, such as HIV [178]. Indeed, pre-clinical studies have proven the efficacy of *Lm* to induce powerful anti-tumoral immunity against a broad range of tumor specific antigens [166,179]. *Lm*-based cancer vaccines are now undergoing clinical trials for several cancers including pancreatic cancer, cervical cancer, osteosarcoma, colorectal cancer, prostate cancer, lung cancer, and more [166,179,180,181,182,183]. However, most of the pre-clinical studies and clinical trials have used i.v. delivery for *Lm*-based cancer vaccines as our understanding of *Lm*-induced immunity has been mainly derived from i.v. infection of mice and questions of whether highly attenuated *Lm* vaccines can be efficacious when administered orally. Oral infection using mouse-adapted *Lm* demonstrated that resident memory CD8 T cells rapidly accumulated in the intestinal mucosa and contributed to protection of a challenge infection [48]. Future studies are warranted to investigate the impact of infection route on CD8 T cell responses in different tissues that could lead to more efficacious vaccine delivery modalities tailored to tumor location. For example, an oral vaccine system may be better suited for protection against tumors that require memory populations residing in gastrointestinal tissues for protection as would be the case for pancreatic, small bowel, or colorectal cancers. On the other hand, i.v. immunization may be better utilized for widely distributed cancers or cancers that have metastasized. Even more intriguing is the notion that *Lm* can be repeatedly administered to boost immune function and this boosting can utilize distinct routes of immunization to tailor the targeting of the immune response as appropriate. 

## 10. Conclusions

*Lm* is a widely utilized pathogen to study T cell biology due to its ability to induce a potent CD8 T cell response and the availability of immunological tools developed in the past decades. Thus, this pathogen has contributed extensively to our general understanding of T cell biology during an immune response. As *Lm* induces potent CD8 T cell responses and CD8 tumor-infiltrating lymphocytes play a critical role in mediating anti-tumoral immunity [184,185,186], *Lm* has become a promising cancer vaccine vector. Dissecting each phase of the CD8 T cell response to *Lm* infection will broaden our understanding of T cell biology in general and contribute to rational vaccine designs. Future studies to understand how the immunization route regulates organ-specific CD8 T cell responses and how these organ-specific CD8 T cell responses may contribute to enhanced protective immunity may further improve T cell-based vaccine development.

## Abbreviations

ActA—actin assembly-inducing protein; Batf3—basic leucine zipper ATF-like transcription factor 3; CCR—C-C motif chemokine receptor; CX_3_CR1—C-X_3_-C motif chemokine receptor 1; DC—dendritic cells; dpi—days post infection; Ecad—E-cadherin; EEC—early effector cells; HK*Lm*—heat-killed *Lm*; IFN—interferon; IL—interleukin; InlA—internalin A; IRF—interferon regulatory factor; i.v.—intravenous; KLRG-1—killer cell lectin-like receptor G1; LLO—listeriolysin O; *Lm*—*Listeria monocytogenes*; LP—lamina propria; MARCO—macrophage receptor with collagenous structure; MMM—marginal metallophilic macrophages; MLN—mesenteric lymph nodes; MP—mononuclear phagocytes; MPEC—memory precursor effector cells; MZM—marginal zone macrophages; NK—natural killer; SLEC—short-lived effector cells; TCR—T cell receptor; T_CM_—central memory T; T_EM_—effector memory T; Th—T helper; TGF-β—transforming growth factor β; TNF—tumor necrosis factor; T_RM_—resident memory T; VSV—vesicular stomatitis virus.
